# Bio-Inorganic Layered Double Hydroxide Nanohybrids in Photochemotherapy: A Mini Review

**DOI:** 10.3390/ijms231911862

**Published:** 2022-10-06

**Authors:** N. Sanoj Rejinold, Goeun Choi, Jin-Ho Choy

**Affiliations:** 1 Intelligent Nanohybrid Materials Laboratory (INML), Institute of Tissue Regeneration Engineering (ITREN), Dankook University, Cheonan 31116, Korea; 2 College of Science and Technology, Dankook University, Cheonan 31116, Korea; 3 Department of Nanobiomedical Science and BK21 PLUS NBM Global Research Center for Regenerative Medicine, Dankook University, Cheonan 31116, Korea; 4 Division of Natural Sciences, The National Academy of Sciences, Seoul 06579, Korea; 5 Department of Pre-Medical Course, College of Medicine, Dankook University, Cheonan 31116, Korea; 6 International Research Frontier Initiative (IRFI), Institute of Innovative Research, Tokyo Institute of Technology, Yokohama 226-8503, Japan

**Keywords:** inorganic bio-nanohybrids, clay nanoparticles, phototherapy, combined photochemotherapy, future directions

## Abstract

Clay-based bio-inorganic nanohybrids, such as layered double hydroxides (LDH), have been extensively researched in the various fields of biomedicine, particularly for drug delivery and bio-imaging applications. Recent trends indicate that such two-dimensional LDH can be hybridized with a variety of photo-active biomolecules to selectively achieve anti-cancer benefits through numerous photo/chemotherapies (PCT), including photothermal therapy, photodynamic therapy, and magnetic hyperthermia, a combination of therapies to achieve the best treatment regimen for patients that cannot be treated either by surgery or radiation alone. Among the novel two-dimensional clay-based bio-inorganic nanohybrids, LDH could enhance the photo-stability and drug release controllability of the PCT agents, which would, in turn, improve the overall phototherapeutic performance. This review article highlights the most recent advances in LDH-based two-dimensional clay-bio-inorganic nanohybrids for the aforementioned applications.

## 1. Introduction

Cancer therapies have advanced with the development of novel therapeutic models, such as personalized or precision medicine [1,2,3,4], but they continue to suffer from a considerable number of limitations. One of the most promising therapies, namely, phototherapy [5,6,7], has negligible side effects because of high selectivity and can be used to treat even deep-rooted tumors easily, such as liver tumors [8]. Phototherapy mainly consists of two types of therapies, namely, photothermal therapy (PTT) [9,10,11], which converts light to therapeutic heat energy, and photodynamic therapy (PDT) [12,13,14], which converts light to therapeutic reactive oxygen species (ROS) [15]. With the recent advances in nanotechnology, these therapeutic strategies are often combined to improve the overall efficacy of phototherapy. In addition, recent studies have utilized phototherapies in combination with chemotherapy, which is generally referred to as photochemotherapy (PCT). However, phototherapy suffers from a number of limitations, especially with regard to phototherapeutic agents [16]. In general, near-infrared (NIR) dyes or ROS generators are destabilized due to degradation. Researchers have attempted to improve the stability of phototherapeutic agents through encapsulation technologies, via polymers [17], liposomes [18], or micelles [19].

Although there are several nanotechnological tools available for phototherapy, they still suffer from their own disadvantages, such as low phototherapeutic stability and toxicity. Recent studies have focused on the potential use of clay-based inorganic nanoparticles (NPs) in PCT applications. Clay NPs are clay minerals that are also referred to as sheet-silicates or phyllosilicates and form part of inorganic layered nanomaterials [20]. They have been used in healing agents [21] and as hemorrhage inhibitors [22]. Furthermore, they are used in pharmaceuticals as active ingredients in oral antacids, gastrointestinal protectors, and anti-diuretics, topically as dermatological protectors and anti-inflammatories [23], and in pharmaceutical preparations as disintegrants, diluents, binders, emulsifying agents, thickening agents, anticaking agents, flavor reservoirs, and the delivery modifiers of active agents [24].

Clay minerals are well known for their layered design, comprising polymeric sheets of silica (SiO_4_) tetrahedra that are attached to octahedral sheets of Al, Mg, and Fe(O,OH)_6_ [25]. These layered-type aluminosilicate minerals are formed during the chemical weathering of other silicate minerals that are present on the Earth’s surface (Ismadji et al., 2015). It is difficult to define “clay minerals” because of the multiple meanings of the word “clay.” It can be used to denote a family of minerals or can pertain to a size fraction (0.98–3.9 mm) in soils and sediments. The latter is the most accepted definition. Generally, clay minerals are less than 2 µm in diameter [26]. Therefore, they have been described as micro- and nanocrystalline materials with a plate-like morphology because of the layered polyhedral arrangement in their structures [25].

**Scheme 1:**
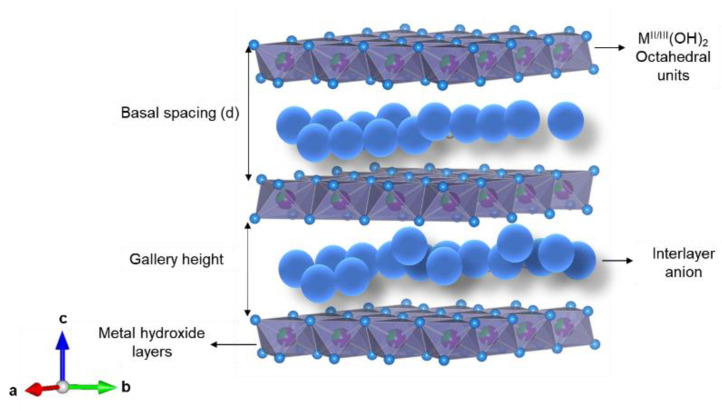
Layered double hydroxides, built from sheets of [M^II^_1−x_ M^III^_x_(OH)_2_]^x+^ octahedral units intercalated with anions (large blue spheres). Each octahedron is composed of a metal cation (M^II^ or M^III^), coordinated with six OH^−^ ligands (small blue spheres).

Even though there are different types of clay NPs (such as anionic and cationic clays), anionic clay, in particular, the layered double hydroxides (LDHs) [27], has special advantages. Most importantly, the chemical composition of LDH clays can be controlled; therefore, the tunability for further functionalization is easier with cost-effective preparation technologies. In general, LDHs are a class of anion-exchange materials with a general chemical formula of [M^II^_1−x_M^III^_x_(OH)_2_]^x+^[A^n−^_x/n_]^x−^·yH_2_O (M: metal, A: anion). The isomorphic substitution of divalent metal cations (M^II^) in otherwise neutral brucite-like M^II^(OH)_2_ sheets with trivalent cations (M^III^) produces cationic charges in the hydroxide layers. In the overall LDH structure, the intercalation of anions (A^n−^) in the interlamellar space compensates for the generated layer charges, as illustrated in Scheme 1.

The biggest advantage of clay NPs is their negligible toxicity at doses predominantly higher than with most other nanomaterials [28]. In addition, their degradation products are biocompatible and biodegradable, compared to most phototherapeutic agents [29]. Moreover, there is substantial evidence that confirms the potential effects of clay-based NPs in several cellular mechanisms, such as proliferation, differentiation, and regeneration [30].

In the case of phototherapy, the chemical stability of common phototherapeutic agents is critical [31]. Clay NPs have the potential to significantly improve the photostability of phototherapeutic agents in several physicochemical reactions, such as in intercalation and ion-exchange reactions, depending on the research strategy.

Although there are several clay-based NP review articles on drug delivery, a specific review on LDH-based hybrids and their PCT applications and recent trends has not been published. For example, Khatoon et al. (2020) and Dong et al. (2020) reviewed the general drug delivery applications of clay materials. However, these reviews did not discuss the use of clay materials in PCT applications [32]. In another review, various applications of two-dimensional (2D) nanomaterials, including photothermal therapy, water evaporation, thermochemical reactions, electrostatic lithography, catalysis, light-driven actuation, photothermal electrodes, energy storage, wearable heaters, and wound healing have been discussed [33]. A similar, previously published review (Chimene et al., 2015) also highlighted similar studies but only focused on general 2D nanomaterials for biomedical applications [34].

The application of clay-based NPs in PCT has several advantages because of their innate physicochemical functions, pore volume, internal surface properties, and structural and exchangeable cations [35,36]. The properties of these materials could be varied depending on the type of clay mineral and its structure. The basic chemistry of novel inorganic hybrid structures is also of great interest. Therefore, it is important to review the most recent developments in clay-based bio-inorganic nanohybrids acting as phototherapeutic and photochemical agents, along with their hybridization efficiency, since it impacts the therapeutic outcome. We believe that a fundamental as well as deep understanding would be highly beneficial to establish their existing issues or benefits so that scientists can improve their overall effects to maximize the therapeutic outcome.

The present review summarizes the recent trends in LDH-based bio-inorganic nanohybrids in terms of PCT applications and their chemical and biological aspects, along with future perspectives. We hope that bringing attention to these fundamental aspects of LDH-based clay hybrids will increase the number of prospective applications.

## 2. Inorganic Bio-Nanohybrids

### 2.1. Improvement of Photochemotherapy Using Inorganic Bio-Nanohybrids

The reason that clay-based NPs are selected for PCT applications lies in their exceptional biocompatibility, even at higher doses, and their pH specificity, which makes them suitable as efficient phototherapeutic delivery systems for effective cancer therapy. Most importantly, the phototherapeutic agents can be safely loaded, without photo-bleaching or sudden disintegration, into the interlayer nano-spaces of the clay NPs (Scheme 1).

### 2.2. Production of Inorganic Bio-Nanohybrids

**Scheme 2:**
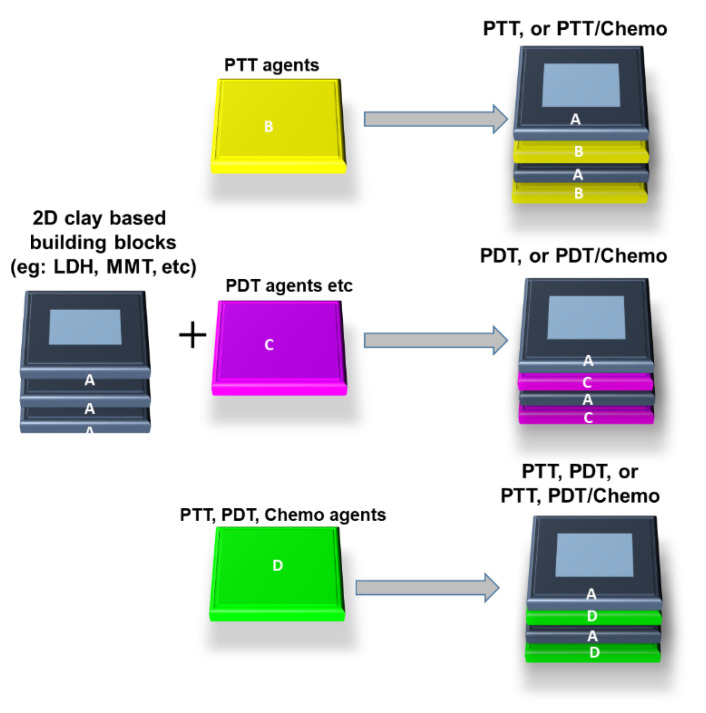
Novel two-dimensional bio-nanohybridization strategies for photochemotherapy applications. (A: 2D clay-based building block that is primarily LDH; B: PTT agents; C-PDT agents and D: PTT, PDT, and chemo agents).

Bio-inorganic nanohybrids can be produced through a “convergence” approach that incorporates the properties of the parent materials via a hybridization process. In brief, “convergence” is a strategical approach for coupling different materials to achieve better results than with their individual forms (Scheme 2). Although such approaches were initially applied in the field of energy research, they have since spread to the drug delivery and tissue engineering fields [37].

The key mechanism for producing bio-inorganic nanohybrids is by means of “2D lattice engineering” [38,39,40,41,42,43,44], where the 2D known host building blocks are stacked with the known inorganic, organic, or biomaterials needed for producing hybrid materials. The host materials used for studying hybrid materials are generally LDH and montmorillonite (MMT) NPs because of their excellent biocompatibility, tuneability, and pH sensitivity [45,46,47,48,49,50].

There are several lattice engineering reactions, such as intercalation, ion exchange, propping open, co-precipitation, exfoliation-reassembling, calcination reconstructing, and pillaring to make hybrid nanoparticles for phototherapy. In particular, PCT agents can be loaded in the interlayer nano-spaces of 2D lattices using the above-mentioned lattice engineering tools [51].

### 2.3. Photochemotherapy

Recently, PTT and PDT have been extensively researched for oncological applications. With nanotechnology-aided phototherapeutic agents, it is possible to improve the overall performance of PCT. However, PCT suffers from numerous problems, such as fast degradation and photo-bleaching. Therefore, stabilizing PCT agents inside the hybrids are of great relevance, which is critical to achieving an optimal therapeutic outcome. The interlayer nano-space and outer surface of clay NPs are highly functional since they are enriched with surface hydroxyl functional groups [35,52,53,54,55,56,57,58,59,60], which can easily be modified through covalent conjugation or hydrogen bonding. Some of the recent LDH-based inorganic hybrids are listed in Table 1.

## 3. Photothermal Therapy and Photothermal/Chemotherapy

The PTT and PTT/chemotherapy (PTT-Chemo) applications of bio-inorganic nanohybrids have been extensively researched. Among them, the LDH-based applications are highlighted in the present review, with a focus on recent developments in the field (2020–2022).

### 3.1. PTT

**Figure 1:**
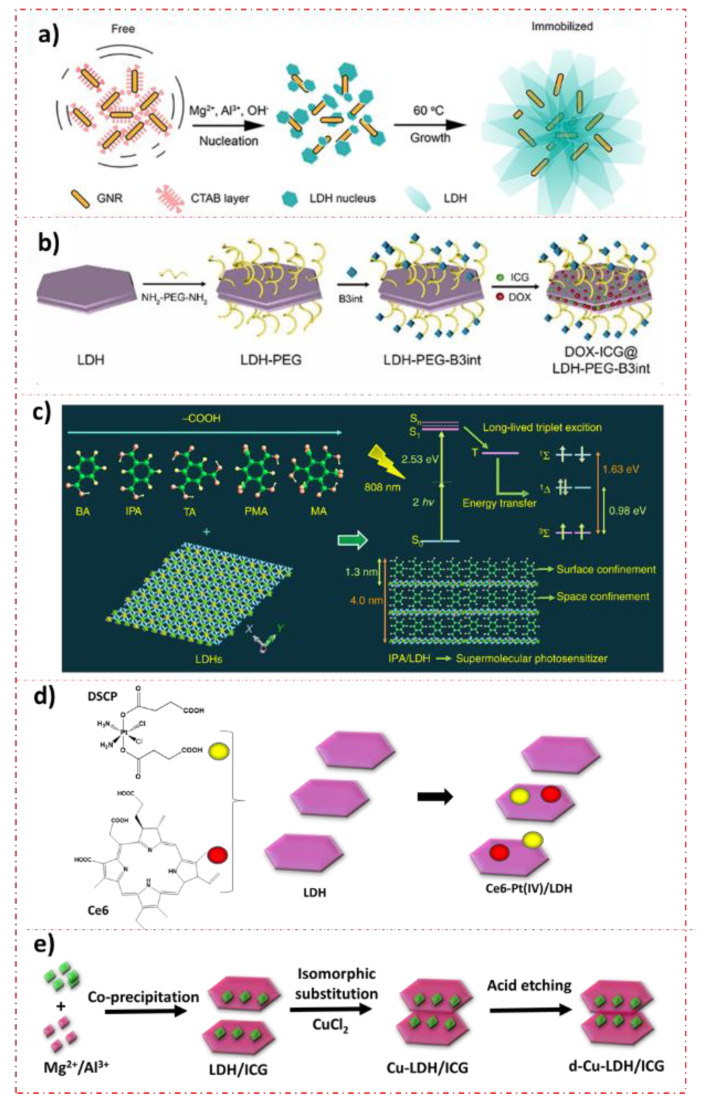
(**a**) Synthesis scheme of gold nanorods (GNR)@layered double hydroxide (LDH) (copyright from ACS-2019). (**b**) Novel two-dimensional (2D) bio-nanohybridization strategies for photochemotherapy applications. Schematic illustration of the synthesis of doxorubicin (DOX)-indocyanine green (ICG) and LDH-PEG-B3int((arginine-tryptophan-(D-arginine)-asparagine-arginine)) nanoparticles (copyright from Elsevier, 2019). (**c**) Illustration of nanohybrids as two-photon photosensitizers for ^1^O_2_ generation. Schematic representation of the LDH host and five aromatic RTP guest species. Two-dimensional-confined long-lived triplet excitons can function as photosensitizers to achieve efficient ^1^O_2_ generation under 808 nm near-infrared laser power (copyrights under Creative Commons Attribution 4.0 International License, *Nature*, 2018). (**d**) Overview of the strategy to co-deliver the Pt(IV) prodrug, DSCP (c,c,t-(diamminedichlorodisuccinato) Pt (IV), and the photosensitizer, Ce6 (chlorin e6), using LDH nanoparticles. (**e**) Synthetic steps involved in the development of trimodal photothermal therapy/photodynamic chemotherapy using d-Cu-LDH/ICG nanohybrids.

Gold NPs, and gold nanorods (GNRs) in particular, have frequently been used for PTT applications [72,73,74,75,76,77]. The photothermal conversion efficiency (*η*) of GNRs are crucial. This can be tuned by increasing the aspect ratio and forming a core−shell structure with LDH NPs (Figure 1a). The interaction between GNRs and LDHs can induce electron deficiency on the surface of GNRs, generating therapeutic heat more efficiently. Therefore, the *η* value of new hybrid NPs can reach 60% when irradiated at 808 nm of laser power, enhancing the *η* value substantially. GNRs are coated with cetyl trimethyl ammonium bromide (CTAB), which can be replaced during the synthesis process. In addition, GNRs maintain a good dispersion in LDHs. This biocompatible core−shell composite GNRs@LDH can be applied to photothermal, antibacterial, tumor therapy, and bio-imaging capabilities [78].

Similarly, a series of novel CoFe-based PTT agents (CoFe-x) have been produced by heating CoFe-LDH nanosheets (NSs) at temperatures (x) between 200 and 800 °C under an argon environment. The produced hybrids differed based on their particle size, non-stoichiometry with cobalt deficiency, mixed electronic configurations, and band structures. Among them, the CoFe-500 product, which had the highest Co^2+^ defect, was the most efficient PTT agent when irradiated under 808 nm of laser power. Experiments and density functional theory calculations confirmed that Co^2+^ defects could modify the electronic structure of CoFe-x and could reduce the band gap, thereby increasing the non-radiative recombination rate and the PTT effects. In vitro and in vivo experimental results proved that CoFe-500 is an excellent PTT agent and can be traced using several imaging techniques, including NIR, magnetic resonance imaging (MRI), and photoacoustic (PA) techniques [61].

### 3.2. PTT-Chemotherapy

In PTT-Chemo approaches, the chemotherapy drug is either chemically or physically incorporated into the LDH NPs to achieve the maximum therapeutic efficacy with PTT. For example, a multifunctional LDH-nanohybrid system was developed for PTT-Chemo to specifically target the *α_5_β_3_* integrin receptors on cancer cells. Hence, arginine-tryptophan-(D-arginine)-asparagine-arginine (B3int) was loaded in the LDH through an EDC/NHS reaction, as shown in Figure 1b. The obtained product was further loaded with indocyanine green (ICG) and doxorubicin (DOX) to form the final product, LDH-PEG-B3int NPs, with approximately 19% drug loading efficiency and a remarkable *η* value of approximately 25%. In addition, the drug release behavior was dependent on the pH and NIR power. In vitro and in vivo studies confirmed that the anti-tumor efficacy was significantly better for nanohybrids compared to their individual components. This result was experimentally determined using *α_5_β_3_* integrin-positive B16 (murine tumor cell line) [62].

Designing an effective theranostic system with tumor micro-environmental responsibility has several potential advantages, including better diagnosis and improved tumor homing characteristics, which would enhance the clinical output. To achieve this goal, 2D hybrids were synthesized by doping MgAl-LDH with functional ferrous ions, which were then loaded with DOX to form Fe-LDH/DOX NPs. The hybrid NPs had MRI (magnetic resonance imaging)-guided synergistic chemotherapy/PTT for breast cancer treatment. The doping of Fe-LDH/DOX with ferrous ions enabled a strong photo-induced heating ability with a high *η* value of approximately 46%, which could be combined with DOX to have synergistic PTT and chemotherapeutic benefits. In addition, the in vitro pH-dependent degradation behavior and T2-weighted MRI effect revealed that the as-prepared Fe-LDH/DOX is sensitive to an acidic tumor microenvironment. Most importantly, the growth rate of tumors in 4T1-bearing mice was effectively suppressed after treatment with hybrid NPs. These results showed that the metal doping of LDH NPs could introduce a novel approach for fabricating an LDH NP-based nanotheranostics platform with advanced diagnostic and therapeutic performance [63].

**Scheme 3:**
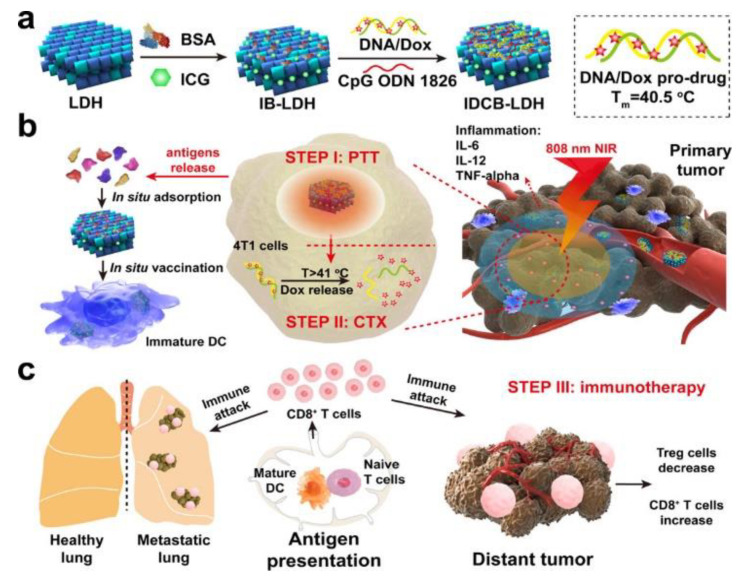
Schematic representation of a multifunctional inodocyanin green (ICG)/Dox/DNA/CpG/BSA-LDH (IDCB)-layered double hydroxide (LDH) nanomedicine. (**a**) This hybrid nanomedicine was constructed via initial coating with bovine serum albumin (BSA) and then orderly loading with indocyanine green (ICG), the doxorubicin (DoX)/DNA prodrug, and CpG ODN (oligodeoxynucleotide) 1826. (**b**) IDCB-LDH, with 808 nm NIR irradiation, heats the tumor tissues and releases DoX at a temperature above 41 °C, yielding anti-tumor effects through efficient photothermal therapy and the subsequent CTX (chemotherapy), which then results in sufficient tumor antigens and stimulates the secretion of pro-tumor cytokines. The residual ICB-LDH in the tumor tissue further acts as a nano-adjuvant and adsorbs the in situ-generated tumor antigens to mature and stimulate DCs (dendritic cells). (**c**) Mature DCs activate naïve T Cells in the dLNs (draining lymph nodes) and induce potent cytotoxic T lymphocytes (CTLs), namely, CD8+ T Cells. The CTLs subsequently migrate to the distant tumor tissues and the metastatic tumor nodules in the lung to eliminate the tumor cells. The CTLs increase, while the regulatory T cells decrease, in the distant and metastatic tumors (Reprinted/adapted with permission from Ref. [64]. Copyright 2019, American Chemical Society).

It is well known that both cancer recurrence and metastasis are global challenges. However, bimodular strategies, such as PTT-chemotherapies, are only successful in a limited number of cases. To improve these bimodular strategies, a multifaceted nanomedicine has been rationally developed by loading three FDA-approved therapeutics, namely, ICG (for PTT), DOX (for chemotherapy), and CpG (for immunotherapy) into the LDH NPs, to eliminate the recurrence and metastasis of invasive breast cancer (Scheme 3). According to an experimental analysis conducted on a 4T1 breast cancer model, the primary tumor tissues were completely eliminated. Therefore, no further recurrence of cancer and lung metastasis was observed. In addition, distant tumors were inhibited because of the improved immunity. Most importantly, the low drug dosage of FDA-approved drugs (ICG, DOX, and CpG) are the highlight of these nanohybrid systems and are expected to further improve the clinical outcome [64].

## 4. PDT and PDT/Chemotherapy

In recent years (2020–2022), PDT, whether used individually or combined with either PTT or chemotherapy, has not been widely explored. This is mainly due to the lack of proper lattice engineering strategies. However, there are only a few studies that have utilized LDH NPs as a base material for such applications. These studies will be explored in the following sections.

### 4.1. PDT

ROS (reactive oxygen species) have garnered considerable attention, not only in the field of catalysis research but also in biological studies because of their strong oxidizing properties [79,80,81]. In particular, the photosensitizers in PDT applications have four major properties, namely, the capability of generating singlet oxygen (^1^O_2_), long-wavelength absorption, good hydrophilicity, and biocompatibility. To achieve these properties in a single system, an NIR-responsive supramolecular photosensitizer-based isophthalic acid and LDH have been integrated as an efficient two-photon PDT nanohybrid system (Figure 1c). The ^1^O_2_ quantum yield of the nanohybrid reached 0.74. The in vitro anti-cancer efficacy on HeLa cells revealed that the approximate IC_50_ value was 0.153 μg mL^−1^. Furthermore, in vivo experiments on a HeLa tumor-bearing animal model confirmed that the nanohybrids were able to achieve deep tumor penetration when they were irradiated under 808 nm laser power, resulting in complete tumor eradication without any side effects. This proof-of-concept analysis clearly indicated that ultra-long-lived triplet excitons can contribute as two-photon-activated photosensitizers for efficient ^1^O_2_ production [65].

### 4.2. PDT/Chemotherapy

In this approach, chemotherapy drugs are combined with the PDT system to produce a desired nanohybrid, which is expected to improve the overall performance of the therapeutic regimen. For example, such a nanohybrid was made by assembling the ratiometric co-loading of Pt(IV) prodrug and Ce6 (PDT agent) into LDH NPs (Figure 1d). The as-made nanohybrids induced anti-cancer effects on cisplatin-resistant human cancer cells (A2780cisR cells) at nano-molar IC_50_ values via a combined PDT-Chemo mode. The in vitro experiments of Ce6-Pt(IV)/LDH on cisplatin-resistant and sensitive cells showed predominant apoptosis, compared to the untreated cells. Especially in A2780cisR cells, nanohybrids induced high early apoptotic cells (approximately 23%) and necrotic cells (approximately 39%) [66].

PDT-Chemo efficacy is mainly dependent on the endogenous H_2_O_2_ concentration. However, it could be varied and is not effective for improved efficacy. Therefore, a self-supplied H_2_O_2_-enhanced PDT-Chemo strategy was achieved by engineering 2D sheet-like nanoparticles, in order to catalyze the molecular reactions. The nanocatalyst was produced by assembling ICG and Fenton reaction catalyst Fe^2+^ ions into two-dimensional ultrathin LDH NPs. Under NIR irradiation, ICG generates cytotoxic singlet oxygen (^1^O_2_), which cripples malignant cells, and superoxide radical (O^2−^), which is further converted to H_2_O_2_ by reacting with intracellular superoxide dismutase (SOD). A sufficient self-supplied H_2_O_2_, together with endogenous H_2_O_2_, is then catalyzed by Fe^2+^ and released from the nanocatalyst to produce a sufficient amount of highly cytotoxic hydroxyl radicals (OH) to induce apoptosis in tumor cells. In vitro and in vivo evaluations demonstrate the remarkable performance of cascade nanocatalyst-mediated PDT-Chemo [51].

## 5. Simultaneous Photothermal/Photodynamic Therapy and Chemotherapy

### 5.1. Photothermal/Photodynamic

It has been suggested that rod/plate-like nanomaterials with an appropriate size exhibit higher and longer-term lysosomal enrichment because the shape plays a notable role in the nanomaterial transmembrane process and subcellular behaviors. Biodegradable LDH-based nanohybrids with CuS nanocomposites (LDH-CuS NCs) were rationally developed through the in situ growth of CuS nanodots on LDH nanoplates. These LDH-CuS NCs exhibited high photothermal conversion, NIR-induced chemodynamics, and PDT efficacies, and real-time in vivo photoacoustic imaging (PAI) of the entire tumor was achieved. In addition, the lysosomal internalized LDH-CuS NCs resulted in higher subcellular ROS in situ, leading to lysosomal membrane permeabilization (LMP) pathway-associated cell death, both in vitro and in vivo [82].

Previously, an iron-manganese LDH NS (200 nm in size) was synthesized to achieve combined PTT/PDT for effective cancer therapy. Methylene blue was loaded into the LDH NPs to enhance the catalase-like activity, which could help the NSs to decompose H_2_O_2_ to O_2_ and overcome tumor hypoxia through O_2_-dependent PDT. The experimental results indicated that these nanohybrids were nearly able to eliminate the whole tumor, as evidenced by the in vivo animal experiments on U14 tumors (squamous mouse carcinoma) [67].

### 5.2. PTT-PDT-Chemotherapy

Although multi-modal therapies can improve the overall efficacy by stimulating the immune community, low tumor accumulation, along with easy immune clearance of the anti-tumor agents, are still difficult to achieve. As cancer cell membranes (CCMs) can have homologous tumor targeting [83], further improvements can be made using multi-modal therapy by complementing the limitations of individual therapeutic modes. In addition, the intracellular uptake of cancer cells can be controlled by photo-induced hyperthermia. CCM-modified-ICG and Abraxane (bovine serum albumin (BSA)-coated paclitaxel (PTX) formulation) were loaded on the LDH NSs to form LIPC NSs (LDH-ICG/PTX-CCM NSs) for the targeted photochemotherapy of colorectal carcinoma (CRC). The CCM-modified NSSs induced effective targeted toxicity and were greatly improved upon laser exposure, synergizing CRC apoptosis. CCM cloaking reduced the uptake of LDH NSs by HEK 293T cells and macrophages, implying mitigation of the side effects and the immune clearance, respectively. The in vivo data further demonstrated that LIPC NSs improved the drug accumulation in tumor tissues and significantly inhibited tumor proliferation under laser irradiation at very low therapeutic doses (1.2 and 0.6 mg/kg of ICG and PTX-BSA, respectively), without severe organ damage. This study clearly demonstrated the importance of CCM coatings on the bio-inorganic nanohybrids, which is critical in achieving the targeted drug delivery and immune-escaping characteristics that can eventually enhance the overall efficacy of drug delivery systems. Furthermore, CCM modifications can also improve the overall hyperthermia by enhancing the cancer cellular uptake of LIPC NSs on cancer cells and improving the overall multi-modal cancer therapy [68].

**Scheme 4:**
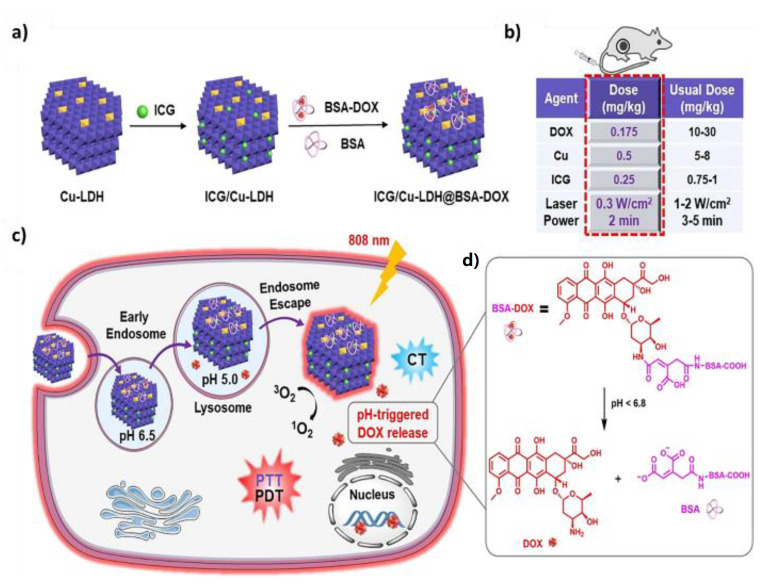
Schematic illustration of a multifunctional indocyanine green (ICG)/Cu-layered double hydroxide (LDH)@BSA–doxorubicin (DOX) nanomedicine. (**a**) Hybrid LDH nanoparticles constructed by making Cu-LDH, loading ICG, and then coating with BSA/BSA–DOX. (**b**) Mice bearing B16F0 tumors are intravenously injected with ICG/Cu-LDH@BSA–DOX and exposed to 808-nm laser irradiation (0.3 W cm^−2^ for 2 min) after 24 h injection, at considerably lower doses (DOX: 0.175 mg kg^−1^, Cu: 0.5 mg kg^−1^, and ICG: 0.25 mg kg^−1^) than those usually used. (**c**) BSA/BSA–DOX-coated ICG/Cu-LDH nanoparticles efficiently accumulate in the tumor site via the EPR effect and facilitate the uptake of tumor cells via the clathrin-mediated endocytosis pathway. In the late endosome, LDH nanoparticles that were neutralized with pumped-in H^+^ ions led to an increased number of Mg^2+,^ Al^3+^, and Cl^–^ ions. An increase in the ionic strength within the endosome drove the water molecules into the endosome, resulting in the osmotic swelling of the endosome and the eventual release of the residual LDH nanoparticles into the cytoplasm. Once internalized by B16F10 cancer cells, ICG/Cu-LDH@BSA–DOX releases therapeutic DOX in response to an acidic environment (pH 5.0–6.5), which synergizes with near-infrared irradiation-induced photothermal therapy (PTT)/photodynamic therapy (PDT) to enable tumor apoptosis. (**d**) The mechanism of pH-triggered DOX release involves amide bond cleavage under a mildly acidic microenvironment of tumor tissues and cells.(Reprinted/adapted with permission from Ref. [69]. 2021, American Chemical Society).

In a previous study, a trifunctional LDH nanohybrid for the combined photochemotherapy (PTT/PDT and chemotherapy) of skin cancer at very low therapeutic doses was explored. This nano-system (ICG/CuLDH@BSA−DOX) was composed of an acid-responsive BSA−DOX prodrug and ICG-intercalated Cu-doped LDH NPs. Thus, ICG/CuLDH@BSA−DOX could have pH-dependent DOX release. In addition, a combinatorial PTT/PDT was achieved under 808 nm laser exposure, inducing dermal cancer apoptosis. In vivo studies confirmed that the ICG/Cu-LDH@BSA−DOX nanohybrid particles were able to satisfactorily ablate the tumor cells when irradiated with a single course of low dosage (DOX: 0.175 mg kg^−1^, Cu: 0.5 mg kg^−1^, and ICG: 0.25 mg kg^−1^) at an 808-nm laser condition of low power at 0.3 W cm^−2^ for 2 min (Scheme 4). This study clearly indicates that rationally designed anti-cancer nanohybrids would be an efficient combination with phototherapy to minimize severe toxic effects and could be useful for translational medicines [69].

In another study, tumor microenvironment (TME)-sensitive nanohybrids based on FeMn-LDH were developed for simultaneous PTT/PDT and chemotherapy applications under single laser irradiation and a power of 980 nm and 0.72 W cm^−2^, respectively, for 5 min. Mesoporous silica and chlorin e6 (Ce6) were covalently coated on the LDH NPs as up-conversion NPs (UCSP) for multimodal imaging-directed cancer therapy. In an acidic environment, FeMn-LDH can be dissolved and will release Fe^3+^ and Mn^2+^ ions to initiate a Fenton-like reaction enabling PTT/PDT/Chemotherapy, along with MRI. Meanwhile, Fe^3+^ can catalytically decompose hydrogen peroxide (2H_2_O_2_) into oxygen (O_2_) and water (2H_2_O), enhancing the PDT guided by UCSP. As a representative non-invasive imaging probe, the up-conversion luminescence can be recovered after the decomposition of FeMn-LDH and can provide high-resolution luminescence for pinpointed PDT. Additionally, the PTT effects of FeMn-LDH can enhance the PDT-Chemo effects. Simultaneous therapy, along with multimodal imaging, can realize the integration of diagnosis and treatment for the efficient theranostic approach, as experimentally proven on the U14 (squamous mouse carcinoma)-bearing tumor model [70].

Similarly, LDH NP-based trimodal inorganic nanohybrids were produced for an efficient breast cancer therapy without any conventional anticancer drugs. NIR-sensitive ICG molecules were integrated on Cu-LDH NPs, which were further acid-etched to induce more Cu defects in the LDH lattice (d-Cu-LDH/ICG). This, in turn, induced trimodal therapy (PTT/PDT/Chemotherapy) under a single laser source (808 nm laser) at a low power irradiation of 2.5 W cm^−2^ for 5 min, at low dosages of 2.5 and 5 mg kg^−1^ (Figure 1e). The as-made nanohybrids could enhance both PTT and PDT simultaneously, while PDT-Chemo was achieved with the same nanohybrid system, as confirmed through the in vitro and in vivo experiments on 4T1 cancer cells and a tumor model, respectively. Moreover, d-Cu-LDH/ICG NPs generate hydroxyl radicals in the presence of H_2_O_2_ as Fenton catalysts, which were further improved upon by Cu(II) reduction with glutathione and temperature elevation. As a result, these nanohybrids were able to suppress 4T1 cell proliferation and showed effective inhibition of tumor growth in vivo, via the multifaceted PTT/PDT/chemotherapy approach under mild laser irradiation. This study clearly indicates that the rational synthesis of defects in LDH nanohybrids would be highly beneficial in combinatorial approaches, without the need for conventional anti-cancer agents. In addition, due to the combined actions of PTT and PDT, complete tumor eradication can be guaranteed, if treated in the early stages of the disease [70].

## 6. Future Perspectives and Conclusions

Biocompatibility and therapeutic efficacy are the major criteria for any biomedical products, along with affordable prices. Of course, LDH-based hybrids could be an easily reproducible material for PCT applications, as the chemical compositions and functionalization are easy to be controlled than many other clay NPs. In addition, from the various literature studies, it is clear that LDH-based bio-nanohybrids hold good biocompatibility and biodegradability. In a reported in vivo study, it was proposed that intramuscularly implanted LDH tablets in the rat abdominal wall could stay there with no notable toxicity. The tablets were composed of chloride ions intercalated into LDH of magnesium/aluminum (Mg_2_Al-Cl) and zinc/aluminum (Zn_2_Al-Cl), respectively. Post-implantation studies using histology assessment reveal no fibrous capsule development around the implanted sites for both types of LDHs. In addition, there were no inflammatory reactions post-implantation with these biomaterials. The sidestream dark field imaging analysis (which is usually used for real-time monitoring of the microcirculation in tissues) confirmed a good microcirculatory network around the LDH-implanted sites and were well maintained, with good blood flow, without enhancing the the leukocyte’s endothelial adhesion. Four weeks of study revealed that the Mg_2_Al-Cl tablet promoted mainly type-1 collagen, whereas the Zn_2_Al-Cl greatly enhanced type-III collagen [84]. However, when considering the LDH-hybrids, especially when integrating them with novel materials for PCT application, it is necessary to clearly understand their long-term toxicity and pharmacodynamics for the benefit of clinical applications.

We suggest that LDH-based photochemical agents are the most cost-effective photosensitive anticancer nanohybrids. However, there are many challenges, as aforementioned, that still need to be overcome before these techniques can be commercially used. Clay-based inorganic NPs are, in general, safe and biocompatible. However, their hybrids could contain a certain degree of toxicity, especially when they are used for long-term applications. Therefore, the toxicity of newly developed LDH-based hybrids should be thoroughly verified.

The stability of colloidal nanohybrids is also critical, especially in the context of injectable formulations and can be highly dependent on the solvents and media used. At present, it is hard to validly report the “stability” of nanohybrids from the literature, as there are not many solid facts from such studies using various conditions, such as simulating body fluid (SBF) or cell culture media, etc. Future studies must be more focused to cast light on the colloidal stability of novel LDH nanohybrids for injectable PCT applications.

Another aspect is utilizing LDH hybrids for scaffold-based PTT for breast cancer bone metastasis. In general, surgical procedures for breast cancer bone metastases have numerous limitations, such as bone defects and tumor recurrence. In such a case, a nacre-mimetic, mechanically stable graphene oxide/layered double hydroxide/chitosan (GO/LDH/CS) layered scaffold with anti-tumor and pro-osteogenesis activities could be beneficial. When exposed to a NIR laser, the local temperature around the scaffolds could rise to 52 °C within 2 min, triggering anti-tumor activity via apoptosis, through increasing caspase-3 expression. Additionally, GO nanosheets and Mg^2+^ ions could enhance the pro-osteogenesis activity of GO/LDH/CS layered scaffolds. Twelve weeks after scaffold implantation, it was found that the layered macropores in the scaffolds were filled with extracellular matrix and bone tissues. Hence, the integration of a nacre-mimetic architecture and biofunctional materials could be a novel strategy to treat intractable disease-related bone defects [85].

Although LDH hybrids have recently begun to be used in PCT applications, this suggests that it takes time for their commercial applications to be realized. Consequently, it is difficult to draw any conclusions about the clinical aspects at the moment. However, we hope that in the near future, there will be new studies emerging in the LDH-based PCT research fields, with more insights on chemical and biological fundamentals and the toxicological aspects.

In conclusion, we have reviewed recent LDH-based nanohybrids for PCT applications, suggesting that such hybrids may be a potential therapeutic strategy for many deep-seated cancers. Such nanohybrids are possible due to the unique chemical nature of LDH and the tunable chemical composition, interlayer nanospace, and ease of surface modification of LDH. However, more studies (pharmacokinetics, biodistribution, organ toxicity, and histological evaluation) are necessary to understand their toxicological aspects for them to be fully utilized in further applications.

## Figures and Tables

**Table 1:** Various clay-based bio-inorganic nanohybrids in photothermal, photodynamic, photothermal/photodynamic, and chemo-combination therapies.

Clay Hybrids	Phototherapeutic Agent or Chemo Drug Used	Applications	Physico/Chemical Characteristics	Preparation Method	Remarks	Ref.
Co-Fe-LDH	Co	PTT, NIR, MRI, and PAI	The TEM image of CoFe-LDH revealed nanosheets with a characteristic hexagonal morphology and a lateral size of 200 ± 20 nm.	Sintering at 200–800 °C under an Ar atmosphere	Theranostic	[61]
B3int, DOX, and ICG loaded in LDH	ICG and DOX	PTT and Chemo	80 nm sized LDH NPs	LDH NPs were prepared by a hydrothermal method followed by surface modification of the LDH NPs with H_2_N-PEG-NH_2_. The targeting peptide B3int was introduced through an amide condensation reaction and finally, DOX and ICG were loaded using a physical adsorption method	Targeted cancer therapy and PTT	[62]
Fe-LDH	Fe-LDH/DOX	MRI, PTT, and chemo	Photothermal conversion efficiency of 45.67% with good stability and pH sensitivity	Doping of ferrous ions into Fe-LDH and DOX loading through physical methods	In vivo results on 4T1 tumor model proved its theranostic properties	[63]
DOX/ICG-CpG-LDH	ICG and DOX	PTT-Chemo-Immuno therapy	The average particle size (DLS) increased from 84 nm (LDH) to 120 nm (BSA coated-LDH) at the BSA/LDH mass ratio of 5:2	Solution mixing	Theranosis toward breast cancers	[64]
Isophthalic acid (IPA)/LDH nanohybrids	Isophthalic acid	PDT	The tapping mode AFM image demonstrates ~50 nm diameter and ~4 nm thickness of IPA/LDH, confirming the formation of ultrathin nanosheets	Co-precipitation method	Combined PDT/chemotherapy	[65]
Ce6/Pt(IV)-LDH	PDT/Chemo	Combined PDT and Chemo	Average hydrodynamic size for LDH is 52 ± 2 nm; the size increases to 150 ± 7, 184 ± 3, and 2633 ± 685 nm for Ce6-Pt(IV)/LDH with the Ce6:Pt(IV) of 0.81, 1.92, and 4.99, respectively	LDH was prepared by hydrothermal method and the Ce6 and Pt(IV) were loaded by anion-exchange reaction	Combined PDT and Chemo approach cisplatin-resistant human cancer cells	[66]
Fe-Mn-LDH	Methylene blue	PTT/PDT	It has 2D nanosheet structure with particle size of about 200 nm and thickness of 2.8 nm comprising 2–3 layers with good dispersibility in water and cell culture medium, with an average hydrated particle size of about 220 nm/zeta potential of −25.4 mV in water and exhibits good stability in both water and cell culture medium	Co-precipitation method	Improved efficacy on U14 tumor model	[67]
LIPC^[a]^ nanosheets	CCM, ICG, and PTX loaded with LDHs	Combined PDT and Chemo	TEM images reveal that LI possessed a typical hexagonal plate-like morphology, with the particle lateral dimension in the range from 50 to 100 nm. After loading PTX-BSA/BSA and coating CCM, LIPC displayed a core–shell structure with a shell of 6–10 nm in length	Physical mixing	Theranostic approach to treat colorectal cancers	[68]
ICG/Cu-LDH@BSA−DOX	ICG, Cu, and DOX	Dual phototherapy and imaging	Hydrodynamic diameter and the zeta potential of Cu-LDH NPs in deionized water were measured to be 58.4 ± 2.5 nm and 37.0 ± 0.6 mV, respectively. The hybrids were in the range of ~200 nm	Physical mixing	Theranostic applications at lower dosage	[69]
Fe-Mn-LDH	Ce6 and mesoporous silica	Trimodal therapy	The Ce6 and silica coating on UCNPs@Ce6@mSiO2 (UCS) possesses an average diameter of 47 nm. The pure FeMn-LDH exhibits a 2D ultrathin structure and hexagon structure comprising several layers with the size of ≈100 nm. After anchoring with UCSP NPs, it can be observed that UCSP can be anchored on the FeMn-LDH nanosheets with an increased size of ≈200 nm with good monodispersity. The DLS particle sizes of UCSP, FeMn-LDH, and UCSP-LDH are 58.8, 130, and 230 nm, respectively with excellent stability in both water and cell culture medium after two days’ standing	Physical mixing	The trimodal theranostic approach was validated on a U14-bearing tumor model	[70]
d-Cu-LDH/ICG	d-Cu-LDH and ICG	Trimodal therapy	Cu-LDH, d-Cu-LDH, and LDH/ICG NPs showed typical plate-like morphology with almost the same particle size distribution with the average hydrodynamic particle size from 25.9 ± 1.1 to 38.8 ± 1.8 nm and zeta potential of around 34–35 mV	Intercalation of ICG into the interlayers of d-Cu-LDH	PTT and PDT-Chemo	[71]

Abbreviations: LDH, layered double hydroxide; PTT, photothermal therapy; NIR, near-infrared; MRI, magnetic resonance imaging; PAI, photoacoustic imaging; B3int, arginine-tryptophan-(D-arginine)-asparagine-arginine; DOX, doxorubicin; ICG, indocyanine green; PDT, photodynamic therapy; CCM, cancer cell membrane; PTX, paclitaxel; LIPC, LDH-ICG/PTX-CCM; BSA, bovine serum albumin; CDT, chemodynamic therapy; PTT, photothermal therapy; IPA, Isophthalic acid; Ce6, chlorin e6; 4T1, breast cancer cell line derived from the mammary gland tissue of a mouse BALB/c strain; U14- squamous mouse carcinoma.

## Data Availability

Not applicable.
